# Introducing a Novel Method of Intravascular Adeno-associated Virus-mediated Gene Delivery

**Published:** 2018-02-27

**Authors:** ZH Fazal, K Hosaka, FP Manfredsson, BL Hoh

**Affiliations:** 1 Department of Neurosurgery, University of Florida, Gainesville, FL 32610, USA; 2 Department of Translational Science and Molecular Medicine, College of Human Medicine, Michigan State University, Grand Rapids, MI, USA

**Keywords:** AAV, Intravascular, Viral vector, Gene delivery, Gene therapy, Artery, Vascular disease

## Abstract

**Introduction:**

Adeno-associated virus (AAV) has shown therapeutic potential as a viral vector in various studies of gene therapy. However, research on its use in targeting intravascular cells in a localized manner is lacking. We introduce a novel method to deliver various AAV serotypes intravascularly and examine their efficiency in transducing cells of the murine carotid artery.

**Objective:**

The study aimed to examine the transduction efficiency of AAV-mediated gene delivery in cells of the murine carotid artery both with and without a fully-formed aneurysm. Results of infection were visualized with green fluorescence protein (GFP) reporter gene.

**Methods:**

Naïve murine carotid artery or experimentally-induced murine carotid aneurysm was ligated distally and proximally. A small incision was made and 5 uL AAV2, AAV5, AAV8, or AAV9 was microsurgically injected and allowed to incubate for 30 min. Incision was closed and tissue was excised three weeks following AAV injection. Carotid artery or aneurysm tissue was excised and fixed in 4% paraformaldehyde solution. On both naïve carotid artery tissue and aneurysm tissue, GFP was visualized by immunofluorescence using antibody against GFP.

**Results:**

Three out of four serotypes of AAV successfully transduced cells within both the murine aneurysm tissue and the naïve carotid artery tissue. AAV5- and AAV9-transduced aneurysm tissue showed the greatest presence of GFP, with AAV8 showing less overall fluorescence. AAV2 showed no fluorescence.

**Conclusion:**

AAV-mediated gene delivery is an effective way to transduce cells intravascularly with a transgene of interest. Our method can be generalized across a wide variety of studies to further research or treat other vascular disease.

## Introduction

Adeno-associated viruses (AAVs) have proven to be an effective method of gene delivery in various animal studies and human clinical trials. AAV is derived from a non-pathogenic human virus of the *Parvoviridae* family within the *Dependoparvovirus* genus. The viral vector is known to efficiently transduce both dividing and mature non-dividing cells in a targeted area, and the viral construct allows for insertion of a gene of interest, or transgene, in a viral capsid [1,2]. In general, the AAV vector has a transgene packaging capacity of approximately 5 kb of DNA [3]. As the coding sequence of a transgene increases in length, the vectors transduction efficiency decreases [3]. While this may appear as a limitation, dual AAV vectors have been used successfully to deliver larger transgenes [3]. Therefore, utilization of AAV for gene delivery has become more common and is often the preferred method for site-specific transgene delivery [4].

Early research on AAV focused specifically on AAV2, however recent studies have shown that other serotypes have greater transduction efficiency among a wide range of cell types and tissues [5]. AAV5 has been used in mice and is known to transduce cells of the retina and liver effectively [4]. AAV8 has been shown to perform well in liver and murine muscle transduction [5]. Finally, AAV9 has been shown to perform successfully in the widest range of tissues including the brain, muscle, heart, retina, and lung [2,4]. In a study comparing the efficiency of multiple AAV serotypes transducing the same tissue, AAV9 was shown to exhibit the greatest number of green fluorescent protein (GFP)-positive cells followed by AAV5 and AAV8, which showed moderate levels; AAV2 showed the lowest level of GFP-positive cells. AAV2 has also been implicated as the least stable of the 12 serotypes, with AAV5 being implicated as the most stable [6].

In comparison to other viral vectors such as *lentivirus*, retrovirus, or adenovirus, AAV offers major advantages that have led to its increasing popularity and use in clinical trials. AAV renders less toxicity *in vivo* than its counterparts, and animal models subjected to AAV have shown a very low overall immune response [4]. AAV has broader tissue tropism, and is also less likely to undergo random genome integration or become oncogenic, which is commonly a concern when working with *lentivirus* or retrovirus [4,7]. AAV is also known to withstand multiple freeze/thaw cycles and be resistant to dehydration, allowing for more stable and reliable long-term viability [5].

Across many animal studies of vascular disease, AAV is administered systemically such as through tail vein injection or catheter-mediated infusion, requiring a larger volume of viral vector overall [8,9]. AAV has also been used in the brain to study global gene delivery and target multiple areas of the central nervous system [4]. However, little is known concerning efficient methods in studies requiring more localized delivery and aiming to target cells of the internal vasculature.

Here, we introduce a novel method of AAV-mediated gene delivery *in vivo* that targets vascular cell types in murine artery and aneurysm tissue using 4 serotypes of AAV: AAV2, AAV5, AAV8, and AAV9. Here, we show how 4 serotypes of AAV can be administered to target cells of the internal vasculature in a murine model.

## Methods

### AAV vectors

AAV2, AAV5, AAV8, and AAV9 vectors tagged with green fluorescent protein (GFP) reporter gene were created and viral titers were calculated. Viral titers ranged from 3.5–5.0×1012 vg/mL (viral genomes per milliliter).

### Animals

All animal experimentation was performed in accordance with protocol(s) approved by our institution’s Institutional Animal Care and Use Committee and comply with Animal Research: Reporting of *In Vivo* Experiments (ARRIVE) guidelines.

### *In vivo* vector delivery in naïve murine common carotid artery (CCA)

An incision is made by the clavicles and advanced cranially for approximately 1.5 cm. After locating the submandibular gland, a cut is made just below, taking care to avoid brachiocephalic veins. Once the area near the carotid is exposed, an aneurysm clip is attached to the submandibular gland and reflected cranially and slightly to the right. The right sternocleidomastoid muscle (SCM) is dissected; dissection is performed medially first, then tweezers hold the brachiocephalic veins laterally as the lateral aspect of the SCM is exposed. Using the curved part of the tweezers facing downward, a scooping motion isolates the muscle from the medial to the lateral side and a silk suture is used to reflect the SCM laterally. The right common carotid artery (RCCA) is exposed to the level of the bifurcation, taking care to stay parallel to the vessel. A 3-0 silk suture is placed under the RCCA, then a latex cuff is placed below the RCCA to protect the trachea, etc. The suture is positioned between the latex cuff and RCCA to keep the RCCA elevated above the latex cuff. Once the RCCA is exposed, two pieces of 8-0 or 11-0 nylon sutures are placed in the gap between the RCCA and a latex cuff distally and proximally, respectively. The RCCA is tied distally by the 8-0 or 11-0 nylon suture first by a bow knot method. This is done to halt the blood flow temporarily and to create a “pocket” of the RCCA (approximately 0.5 cm in length). The RCCA is kept moist while AAV is prepared in the injector.

A droplet (5–10 uL) of solution containing AAV2, AAV5, AAV8, or AAV9 (3.5–5.0×10^12 vg/mL) with GFP reporter gene is placed on sanitized parafilm. The solution is then aspirated in a micro glass needle attached to a micro injector. A very small incision on the pocket of RCCA wall is made using a 30-gauge needle close to the suture that was tied proximally. Blood is squeezed out from the pocket by tweezers. The glass needle with viral vector is inserted into the pocket of RCCA *via* incision. The solution is then injected into the pocket of RCCA through careful manipulation of micro injector. Once injection is completed, bipolar cauterizer is placed over the incision with the glass needle. Needle is removed from the incision gently, and incision is closed by bipolar cauterization. Once the incision is closed, the pocket of RCCA with viral vector is incubated for 30 min. After the incubation, the distal suture is removed first, followed by the remaining suture. The skin incision is closed with appropriately sized (5-0 or 4-0; following NIH guidelines) monofilament non-absorbable sutures or surgical staples. The animal is placed on a slide warmer/heat pad to assist in appropriate thermoregulation. A single dose of Carprofen (5 mg/kg IP) is administered to provide analgesia twice in 48 h. The suture or staple is removed 14 days after surgery.

### Murine carotid aneurysm model

Murine carotid aneurysms were created in C57BL/6 female mice (Charles River, Wilmington, MA) with a method previously described [10]. Briefly, the right CCA is microsurgically exposed, and 10 U porcine pancreatic elastase solution (Worthington Biochemical Corp, Lakewood, NJ) diluted in 1 mL of saline (Invitrogen, Carlsbad, CA) is applied extravascularly for 20 min. The vessel is then occluded distally to create a stump. Three weeks later, a saccular aneurysm has formed at the right CCA stump.

### *In vivo* vector delivery in right CCA containing fully-formed aneurysm

Three weeks following elastase exposure, a fully-formed aneurysm in the right CCA is temporarily ligated distally and proximally. AAV serotypes are delivered using the same preparation and operational methods in the naïve murine RCCA mentioned above.

### Histological analyses

Three weeks following AAV2, AAV5, AAV8, or AAV9 microsurgical injection, both murine carotid aneurysm tissue and naïve carotid artery tissue was excised and fixed in 4% paraformaldehyde. After 24 h, specimens were either frozen-prepared or paraffin-embedded. For cryosection, murine tissue was transferred into 18% sucrose solution in PBS for 24 h at 4°C and embedded in OCT compound. The vessel samples were then cryosectioned into 5 µm axial slices. Immunohistochemistry (IHC) was performed on sectioned tissues mounted on glass slides. Slides were rinsed in TBST and incubated in 4% horse serum for 1 h and 1:500 anti-GFP primary antibody for 1 h at room temperature. Slides were then rinsed in TBST and incubated at 4°C for 24 h. Slides were rinsed in TBST and incubated in 1:500 Donkey Anti-Chicken IgY H&L (FITC) secondary antibody for 1 h. Slides were then rinsed with TBST and mounted using VectaShield with DAPI stain (Vector Laboratories). Cells were imaged using a Leica dissection microscope with Volocity 3D analysis software and an Olympus IX71 inverted fluorescent scope (Olympus, Center Valley, Pennsylvania, USA) with Image Pro Plus software (Meyer Instruments Inc., Houston, TX).

## Results

**Figure 1:**
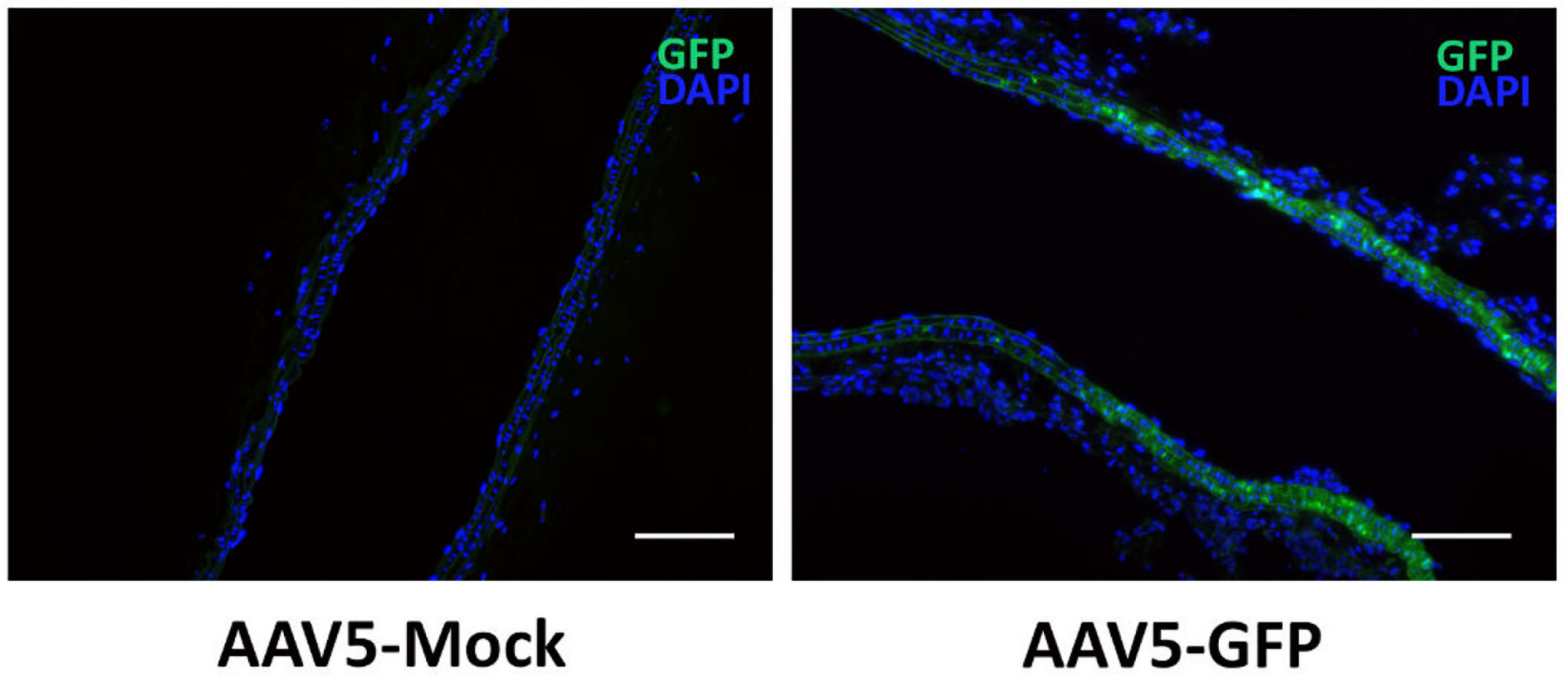
Localized intra-artery viral transduction using AAV5-GFP in mouse common carotid artery. Scale bar=100 m

**Figure 2:**
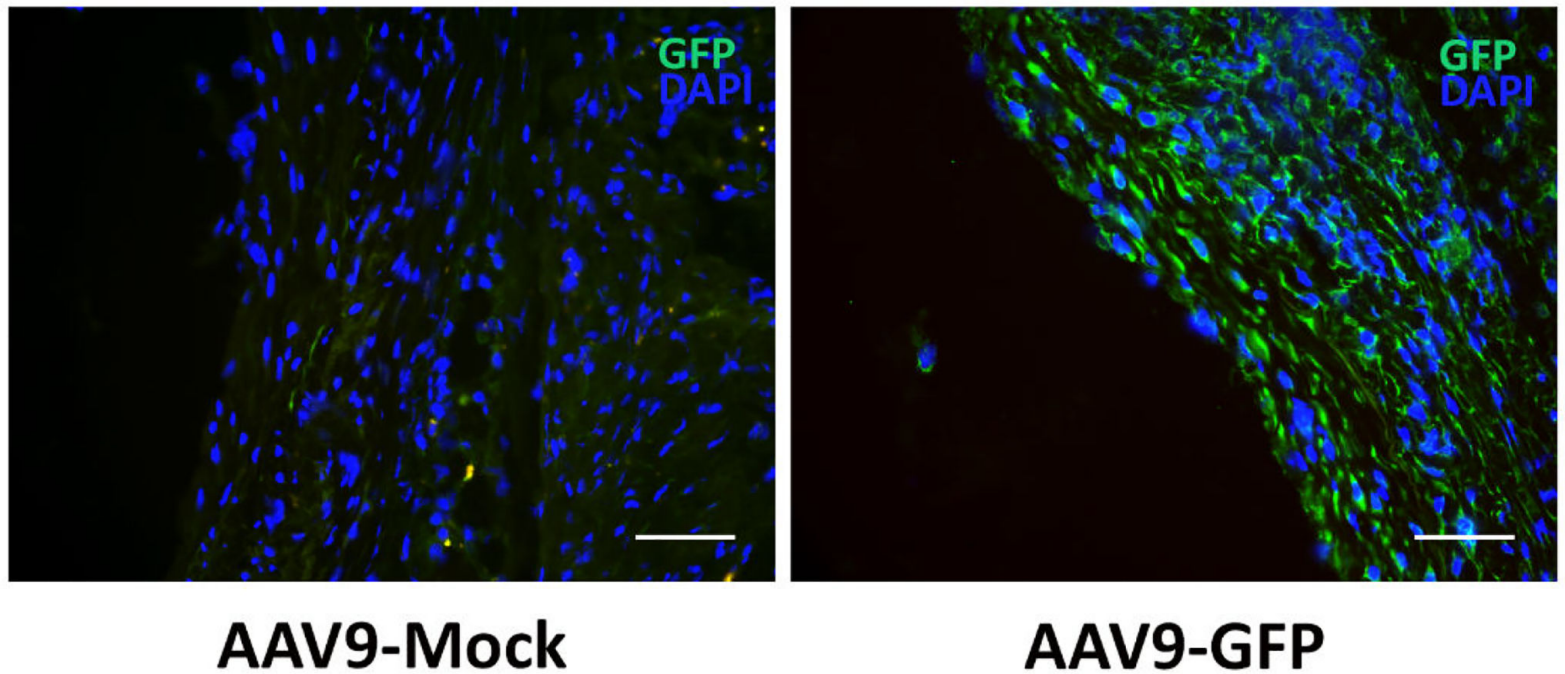
Localized intra-aneurysm viral transduction using AAV9-GFP in mouse. Scale bar= 50µm

Vascular cells in murine RCCA and aneurysm tissue were successfully transduced through use of viral vectors AAV5, AAV8, and AAV9 with GFP reporter gene. Aneurysm tissue subjected to AAV2 showed no GFP expression. Tissue subjected to AAV5 and AAV9 showed the greatest fluorescence, as depicted in Figures 1 and 2 respectively.

## Discussion

Our method allows for the exploration of internal vasculature and can therefore be applied across a wide range of study on vascular disease. Utilizing AAV as a vector for delivery is beneficial in that it can be personalized to create therapy for a disease of interest and as mentioned previously, is least toxic *in vivo* compared to other viral vectors [4]. A therapeutic transgene of choice can be loaded into a viral capsid upon formation of the vector followed by administration locally *via* intra-arterial injection. A gene of interest can also be deleted or added to a target site through use of our method of vector delivery.

Studies sharing similar target tissue and target cell types for whom systemic application has failed can benefit greatly by replicating this novel route of local vector administration.

## Conclusion

We conclude that our AAV-mediated method of gene delivery is an effective way to transduce cells intravascularly. Our method of delivery requires a smaller volume of viral vector per animal and shows low toxicity *in vivo*, allowing it to perform especially well in animal models. Ultimately, this method can be generalized across a wide range of *in vivo* applications focusing on vascular disease including but not limited to atherosclerosis, coronary and peripheral artery disease, blood clotting disorders, and hypertension.
